# A call for Rigor and standardization in plant extracellular vesicle research

**DOI:** 10.1002/jev2.12048

**Published:** 2021-04-27

**Authors:** Marcela Pinedo, Laura de la Canal, Carine de Marcos Lousa

**Affiliations:** ^1^ Instituto de Investigaciones Biológicas Universidad Nacional de Mar del Plata‐CONICET Funes Mar del Plata Argentina; ^2^ Centre for Biomedical Sciences Leeds Beckett University Leeds UK; ^3^ Centre for Plant Sciences University of Leeds Leeds UK

All living cells, including archaea, bacteria, and eukaryotes, release nano‐sized lipid‐bilayer membrane vesicles known as extracellular vesicles (EVs) for reasons ranging from disposal of toxic materials to intercellular communication (Margolis & Sadovsky, 2019). In plant sciences, small EVs were described in the apoplast (the plant extracellular fluid) as early as 1967 (Halperin & Jensen, 1967), around the time of the first observations of mammalian extracellular vesicles (Witwer & Théry, 2019). However, plant EVs were separated from plant apoplastic fluid and visualized by transmission electron microscopy (TEM) only in 2009 (Regente et al., 2009). Since then, plant EVs remained largely ignored until 2017, when two publications furnished strong evidence for their existence and paved the way for studies on involvement in plant‐pathogen interactions (Regente et al., 2017; Rutter & Innes, 2017). Considering the rapid growth of studies of EVs in animals, combined with recently published results on the role of plant EV small RNA in interspecies communication and defense against fungal pathogens (Baldrich et al., 2019; Cai et al., 2018; Roth et al., 2019), the immediate future is likely to bring a great expansion of plant EV research.

Growth of the plant EV field will be optimized if we, as the plant science community, can agree on several matters of rigor and standardization in advance. As a point of reference, the International Society for Extracellular Vesicles (ISEV) has issued consensus guidelines on the nomenclature and minimal information for studies of EVs (Théry et al., 2018). While the general principles of these “MISEV” guidelines are applicable to the plant EV field, specific recommendations on topics such as mammalian EV protein markers do not yet have parallels with plant EVs due to the relatively scarce information that is currently available. Here, we provide some suggestions for the standardisation of plant EV research that we hope will be adopted by scientists working with plant nanovesicles worldwide. Since the existing plant EV literature contains great and confusing diversity of definitions and separation methods, we focus on the nomenclature and characterization of plant EVs.

## 1 NOMENCLATURE: CURRENT STATE OF THE FIELD

How are EVs named? The ISEV consensus recommendation on nomenclature is to use the term ‘extracellular vesicle’ as the ‘generic term for particles naturally released from the cell that are delimited by a lipid bilayer and cannot replicate’ (Théry et al., 2018). Mammalian cells and tissues are known to release diverse EVs into extracellular fluids, and deep physical characterization has been performed following various size‐, affinity‐, and density‐based separations. Hence, mammalian EVs can now be classified into subtypes (exosomes, ectosomes…. etc) according to cellular and subcellular origin, phenotypes, cargo, and perhaps more arbitrarily by size and density ranges (Mathieu et al., 2019). In contrast, clear evidence on plant EV characteristics such as surface markers, sizes and densities is still scarce. Biogenesis pathways are also not well known, although, for example, the term ‘paramural bodies/vesicles’ has been associated with putative exosomes and imaged accumulating in the apoplast at the site of fungal penetration in barley leaves (An et al., 2006). EVs have also been imaged in the apoplast of grapes (Pérez‐Bermúdez et al., 2017).

Because of the current lack of knowledge, it is still difficult to classify plant EVs with the established nomenclature used for animal EVs. Currently, the plant EV literature presents a confusing array of terms, such as nanovesicles, microvesicles, exosomes and exosome‐like vesicles, but unfortunately without strict physical characterization or establishment of biogenesis pathway, a clear nomenclature cannot be established.

## 2 A PROPOSED SOLUTION TO THE NOMENCLATURE PROBLEM

Per convention, EVs are defined as *extracellular* structures naturally released from cells. Mammalian EVs can be separated from different extracellular compartments such as cell culture medium, body fluids (serum, blood, urine, etc.), and the interstitial space of solid tissues. Separation of EVs from plant tissues is a major matter of concern. So far, only a minority of reports have rightly concentrated on purifying EVs from extracellular apoplastic fluids (e.g. after buffer infiltration‐centrifugation of leaves to recover the apoplastic content). However, most published reports have used other methods that are likely to result in tissue/cell rupture, increasing intracellular contamination (Ju et al., 2013; Pérez‐Bermúdez et al., 2017; Yang et al., 2018). These methods involve extraction of fruit juice, naturally contained within vacuoles in several cell layers in the pericarp, by either gently pressing the fruits/plants or harshly grinding them in a mixer/blender. While the first, gentler technique might involve only mild contamination from cellular disruption, the second will definitely result in recovery of not just EVs, but also artificial nanoparticles/microsomes that result from ‘re‐mixing’ of disrupted cellular membranes, as well as native intracellular vesicles that are released when the cell is broken open (György et al., 2011).

**FIGURE 1:**
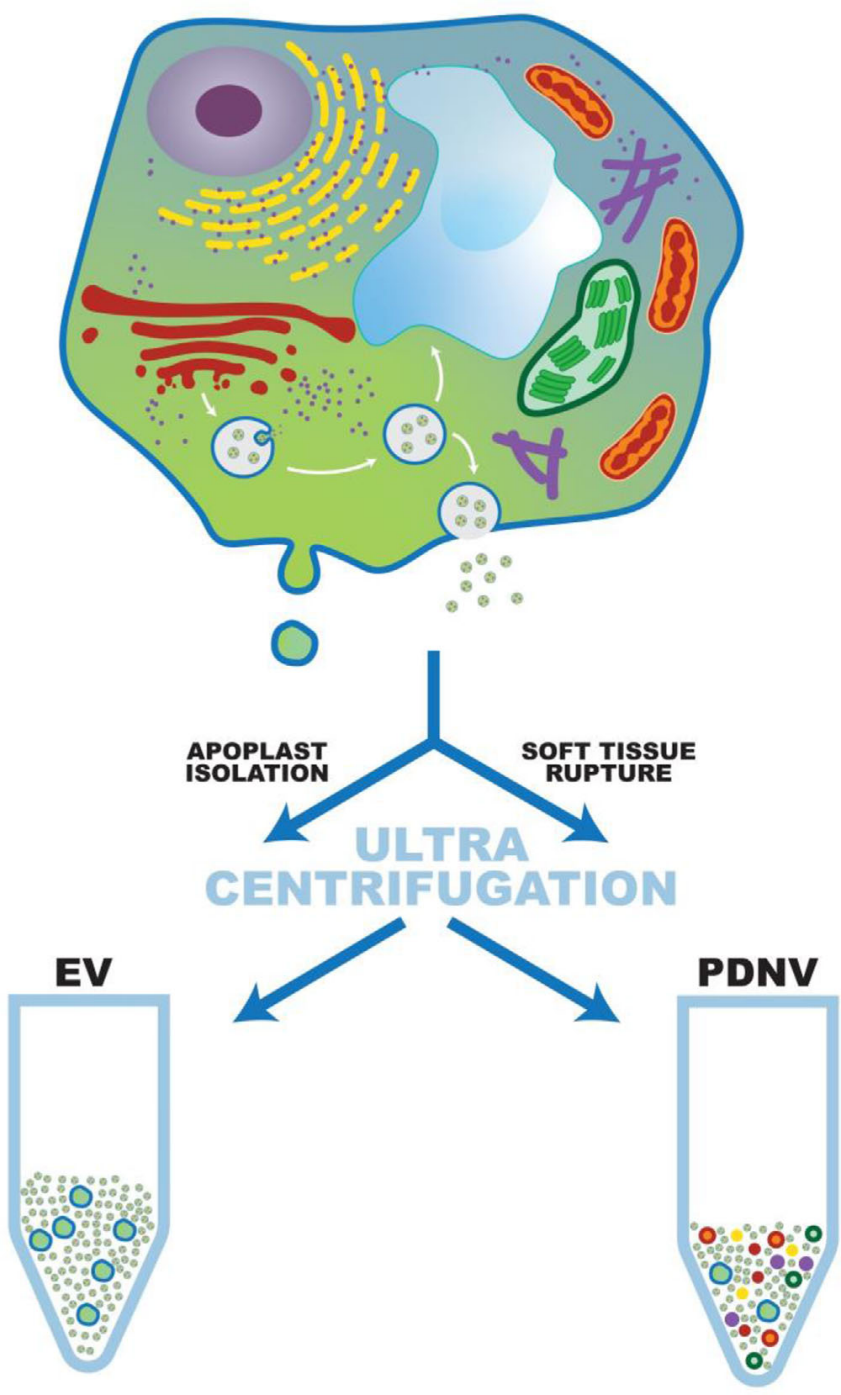
Representation of Plant EV and PDNV isolation procedures. Recovery of apoplastic extracellular fluids is compared with soft tissue rupture. For simplicity, only two types of putative EVs are shown: ectosome‐types (derived from plasma membrane) or exosome‐types (derived from MVBs originating from the Golgi). After differential ultracentrifugation, purification from apoplastic fluids will lead to the purification of natural EVs (exosomes, ectosomes….etc). In contrast, the use of soft tissue rupture will result in “artificial” plant derived nanovesicles (PDNVs) which will contain EVs mixed with intracellular organelle debris such as mitochondria (red), chloroplasts (dark green), ER (yellow), and so forth.

These destructive procedures pose a significant nomenclature issue since there is at present no clear method to distinguish true plant EVs from artificially produced or intracellular vesicles. To make matters worse, diverse mixtures of vesicles obtained by these techniques are often subjected to microfiltration (e.g. with 0.2 or 0.4 μm filters), which positively selects or even produces particles with size ranges similar to those expected for native EVs (30–200 nm diameter). Although some authors do make a distinction by naming these plant‐derived particles ‘exosome‐like nanoparticles’ (ELNs) or ‘plant edible nanoparticles’, it is *essential* that unfamiliar readers are aware of the intrinsic variability in the nature of these artificially produced nanoparticles (mix of vesicles from blended plants) compared with “natural” extracellular nanoparticles (EVs released into apoplastic fluid). Hence, in hopes to standardize the literature, we propose a nomenclature to distinguish artificial vesicles from naturally released EVs. Specifically: (1) the generic term “plant EVs” should be used instead of “exosomes” at least until further characterisation enables a deeper classification by subcellular origin; and (2) “plant‐derived nanovesicles” (PDNV) is suggested for all vesicular fractions obtained from plant tissues when destructive processes are used and when natural release into the extracellular space cannot be established (Figure 1).

## 3 SEPARATION AND CHARACTERISATION METHODS

In addition to adopting the suggested nomenclature, we propose further standardization of plant EV harvesting and separation protocols. The conventional method to isolate apoplastic fractions involves a simple infiltration‐centrifugation protocol of seeds or leaves (O'Leary et al., 2014). The exact composition of the infiltration buffer is important and must be optimized to minimize tissue shearing and contamination with intracellular content. Depending on the species and anatomical features of the tissue to be extracted, a variety of buffers have been used (Regente et al., 2009; Rutter & Innes, 2017; Witzel et al. 2011). For the separation of *Arabidopsis* EVs, a consensus seems to have coalesced around the following composition: 20 mM MES, 2 mM CaCl2, and 0.1 M NaCl, pH 6 (Cai et al., 2018; Rutter & Innes, 2017). In case of the optional addition of protease inhibitors, beta‐mercaptoethanol or detergents during isolation of apoplastic fluids, extreme caution should be taken prior to EV functional studies by washing out these additions. After recovery of apoplastic fluids, various methods can be applied for EV separation, but just as in the broader EV field (Gardiner et al., 2016), differential centrifugation is most widely used. This approach is based generally on established mammalian EV separation protocols but differs slightly by including low‐ speed centrifugations (500 × *g* and 10 K × *g*) followed by ultracentrifugation steps (40 K × *g* or 50 K × *g* and 100 K × *g*). The low speeds allow the removal of debris and large organelles such as mitochondria and chloroplasts. The 40–50 K pellet is generally described as enriched in EVs with a classical size range of 30–200 nm. But the description of the 100 K pellet differs. For EVs isolated from apoplastic sunflower seeds, it was reported as being similar to the 40 K pellet in terms of protein content and size of EVs (Regente et al., 2009), whereas the 100 K pellet from the *Arabidopsis* leaf apoplast (Rutter & Innes, 2017) was described as enriched in uncharacterized smaller particles ranging from 10–17 nm. Consequently, further studies have characterized EVs from either the intermediate (40–50 K) or ultimate (100 K) fractions. This discrepancy and regular focus on an intermediate pelleting fraction seems to be specific to the plant field, since mammalian EV separations have overwhelmingly relied on 10–20 K and 100 K fractions. We refer the reader as well to a summary of good practices for plant EV separation and characterization by Rutter & Innes (Rutter & Innes, 2020). The authors emphasize the importance of distinguishing *bona fide* EV cargo from merely co‐purifying contaminants and point out that plant EV content may vary according to the separation procedure used.

Understanding the outcome of these and other separation methods will require more detailed analysis of yield and purity of EVs, which in turn requires development of reliable markers for plant EVs and potential subtypes (see below). Since a ‘gold standard' separation method thus cannot be proposed at this time, we can make only general recommendations. For example, if differential ultracentrifugation is used, careful consideration of ultracentrifugation steps should be taken, including justification of why a pellet from a particular spin (e.g., 40 K, 50 K, 100 K) was chosen. Per MISEV2018 recommendations, it might also be helpful to study multiple pellets and even the remaining supernatant.

Regardless of which fraction is chosen, differential ultracentrifugation is usually followed by a second purification step to limit co‐contaminations with intracellular components and/or achieve a better separation of EV subtypes (Théry et al., 2018). In the mammalian field, various techniques such as density‐, size‐, precipitation‐ or affinity‐based techniques are being used for separation of EVs from non‐vesicular extracellular material [e.g., (Jeppesen et al., 2019; Kowal et al., 2016; Mathivanan et al., 2010; Sharma et al., 2018) and for review (Gandham et al., 2020; Lee et al., 2019; Li et al., 2019). On the other hand, in the plant field, traditional methods such as density gradients (sucrose or iodixanol) and size exclusion chromatography are being used (Baldrich et al., 2019; Liu et al., 2020; Rutter & Innes, 2017) but others like immunoaffinity remain to be exploited by the plant research community as soon as EV markers become available.

Purifications should then be followed by extensive characterization prior to functional studies. A large range of techniques are available and widely used in the EV field. Amongst them, nanoparticle tracking, TEM and other forms of EM, flow cytometry, nano‐flow, Raman spectrometry or the more recent resistant pulse sensing technique (RPS) are highly recommended to further characterize EVs [e.g., (Kitka et al., 2019; Sharma et al., 2018) and for review (Gandham et al., 2020; Gardiner et al., 2016; Théry et al., 2018) and are starting to be assayed in plants (Cai et al., 2018; Liu et al., 2020; Rutter & Innes, 2017)

## 4 THE NEED FOR PLANT EV MARKERS

**FIGURE 2:**
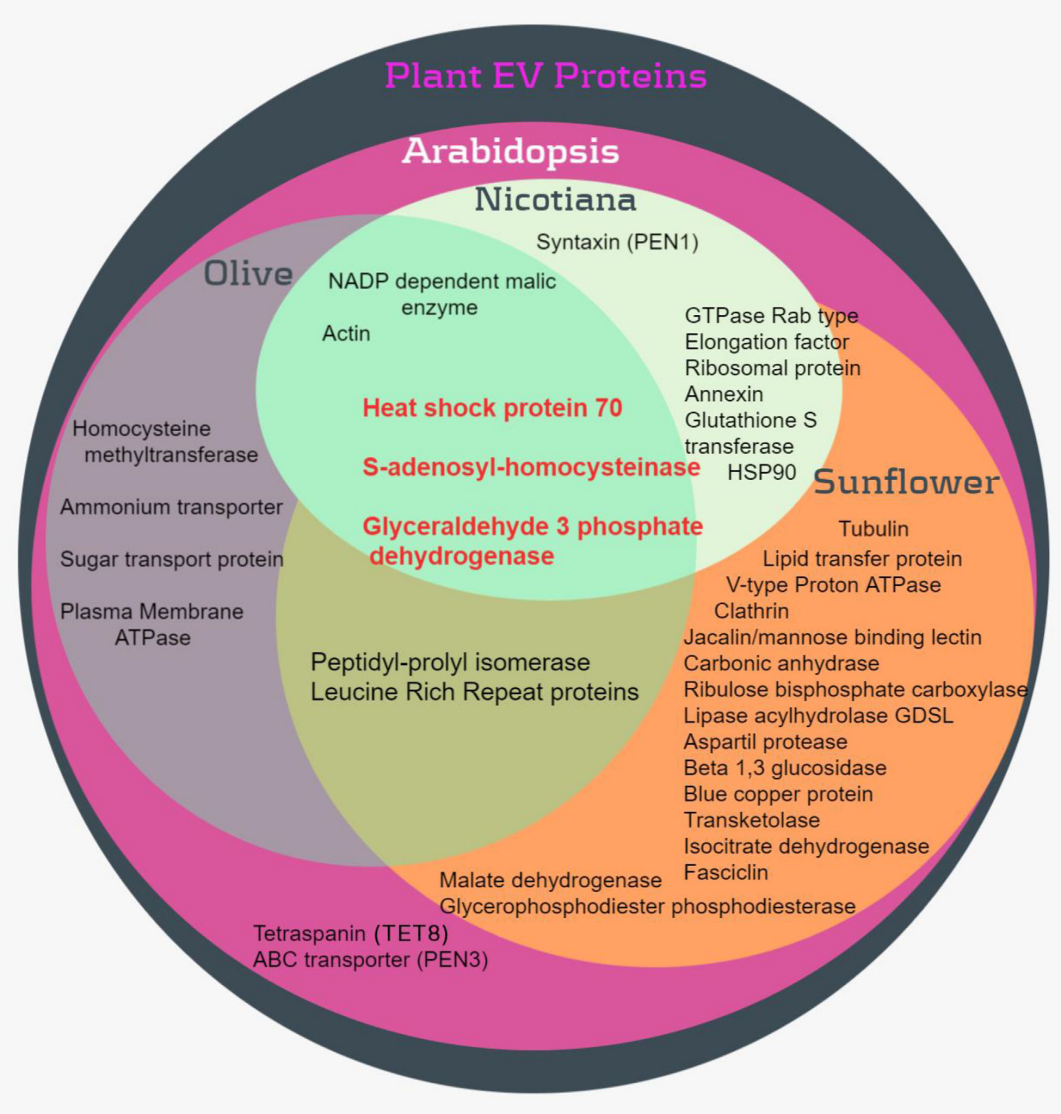
Protein families frequently recovered in Plant EVs. Proteomes of *A. thaliana*, Sunflower, Olive and *N. benthamiana* were compared to retrieve common identities and represent them in a Venn diagram. In red, proteins that are shared between the four proteomes.

However, perhaps the most pressing need is to find suitable and reliable markers of plant EVs. Due to the relative novelty of the field, markers for plant EVs are only beginning to emerge. One reason for this is the relatively small number of plant EV proteomics analyses. Although a few have been published on PDNVs, to our knowledge only four have been reported for apoplastic EVs: olive pollen grains (Prado et al., 2014), *A. thaliana* leaves (Movahed et al., 2019; Rutter & Innes, 2017), sunflower seedlings (Regente et al., 2017), and *N. benthamiana* leaves (Movahed et al., 2019). Comparing them, we identified three protein families commonly found amongst plants (Figure 2) and also present in mammalian EV proteomes according to the Exocarta (http://www.exocarta.org/) and Vesiclepedia databases (http://bigd.big.ac.cn/databasecommons/database/id/6239). They are heat shock protein 70 (HSP70), S‐adenosyl‐homocysteinase and glyceraldehyde 3 phosphate dehydrogenase (GAPDH), which are in the top 100 proteins in animal EVs (http://microvesicles.org/extracellular_vesicle_markers). Furthermore, *Arabidopsis* and sunflower, both with high‐throughput proteomics data, share most similarity, with twenty‐seven proteins in common between them, 10 of which are found in the top 100 proteins in Vesiclepedia.

Proteomic analyses performed on PDNV from squeezed lemons (Raimondo et al., 2015) and other citrus species (Pocsfalvi et al., 2018), high speed blended grapefruits (Wang et al., 2014) and pressed grapes (Ju et al., 2013) also showed some protein families frequently recovered in natural plant EVs. Among them are members of the HSP70, GAPDH, glutathione S‐transferase and Annexin families. The occurrence of common identities between PDNV and EVs is not a surprise based on the contribution of EVs to the PDNV fraction (Figure 1). In fact, a recent comparative analysis of nanovesicles isolated with the same procedure either from disrupted leaf (i.e., PDNV) or the extracellular apoplastic space (EVs) of *A. thaliana* reported similarities and differences among both vesicle types (Liu et al., 2020). Even though they share some characteristics, they differ in size, density distribution and protein content. The study identified 1438 distinct proteins in the *Arabidopsis* leaf nanovesicle and 787 in the apoplast EV samples. The presence of diverse membrane and soluble proteins derived from distinct subcellular origins was observed in EVs and PDNVs, but only the EVs proteome showed a significant relative enrichment of accessions associated with extracellular function and cell wall localization. In fact, only a small portion of the leaf nanovesicle proteins are also found in apoplastic EVs. Notably, the plant EV and human plasma EV proteomes shared more similarities than did the two plant nanovesicle proteomes (Liu et al., 2020).

Clearly, additional plant EV proteomics datasets are needed to start establishing a set of reliable markers. The MISEV2018 guidelines suggest five categories of marker proteins for EV characterization: 1‐ Transmembrane or GPI‐anchored proteins 2‐ Cytosolic proteins recovered in EVs 3‐ Major components of non‐EV co‐isolated structures 4‐ Transmembrane, lipid bound and soluble proteins associated to other intracellular compartments than PM/endosomes and 5‐ Secreted proteins recovered with EVs (Théry et al., 2018). Although the identification of proteins belonging to each of the five suggested categories is unlikely at this stage of plant EVs research, we can propose that an ideal plant EV marker would be enriched in highly purified “natural” plant EVs relative to whole cells. This ideal plant EV marker should also be conserved across plant species, which might be an issue considering the diversity of plants. Based on the current literature, candidates for category 1 are the syntaxin PEN1, the ABC transporter PEN3 (usually involved in fungi penetration resistance) and the Tetraspanin‐8 TET8 (Rutter & Innes, 2017). PEN1 is found consistently in the proteomes from *Arabidopsis* and *N. benthamiana*. Its enrichment in natural EVs was also confirmed via western blot, as a chimeric GFP construct under the native promoter or upon overexpression in both plants (Liu et al., 2020; Rutter & Innes, 2017; Zhang et al., 2020). Although PEN3 is found in natural EVs proteome from *Arabidopsis*, its presence by western blot has not been tested. Finally, TET8 is an interesting candidate because it is structurally similar to mammalian tetraspanin CD63, a proposed *bona fide* marker of a sub‐type of mammalian EVs (Jeppesen et al., 2019; Kowal et al., 2016; Théry et al., 2018). TET8 was proposed as a potential plant marker (Cui et al., 2020) and AtTET8‐GFP construct was enriched in natural plant EVs when expressed from natural promoter or overexpressed (Cai et al., 2018; Zhang et al., 2020). Amongst cytosolic proteins, annexin, Heat shock proteins (HSP70, HSP90) and GAPDH, mentioned above as being found in *A. thaliana*, *N. benthamiana* and sunflower proteomes, are members of category 2. Although a few recent publications have suggested that these proteins might represent specific markers of human EV sub‐types [e.g., Jeppesen et al., 2019; Kowal et al., 2016], it is still unclear for the mammalian field at present. Therefore, their relevance for the plant EV field also needs to be tested. VPS4, a category 2 marker for mammalian EVs and a member of the ESCRT complex, was not detected in natural plant EVs, even upon overexpression (Zhang et al., 2020). In contrast, native Pattelin‐1 and ‐2 (PATL‐1 and PATL‐2), two cytosolic proteins recruited to membranes and probably involved in membrane‐trafficking events, were found enriched in natural EVs and could therefore be considered as putative specific markers (Rutter & Innes, 2017). Members of other categories 3–5 have not yet been characterized in plant EVs. Further studies are clearly essential to identify a *bona fide* plant EV marker. It is recommended that these markers are detected natively rather than upon overexpression which could affect the protein content of EVs. Unfortunately, most antibodies raised against putative plant candidates mentioned above are not commercially available yet. Therefore, at present, the field relies mostly on cooperation and material sharing between plant scientists to push forward the discovery of ideal “universal” plant EV markers.

## 5 PLANT EV FUNCTION: A WORLD OF POSSIBILITIES AWAITS

The importance of establishing proper plant EV nomenclature, separation, and characterization becomes especially obvious when we consider what is at stake: what is already known about the functions of plant EVs and the possibilities that these functions may open for both plant and human health. A clear EV role in plant infection and immunity has become apparent (Cui et al., 2020; Rybak & Robatzek, 2019). EVs have been shown to accumulate at the site of fungal penetration, and their release was stimulated upon infection or treatments mimicking this process (Meyer et al., 2009; Nielsen et al., 2012; Rutter & Innes, 2017). Evidence that plant EVs are taken up by fungal cells and transfer small RNAs to silence fungal virulence genes has been reported (Cai et al., 2018; Regente et al., 2017). Interestingly, the role of a significant population of EV small and tiny RNAs is also emerging (Baldrich et al., 2019). In addition to their involvement in plant defense, data also suggest that EVs could be vehicles for the secretion of cell‐wall related proteins, which would execute a particular role in plants compared to mammals: the remodelling of the cell‐wall (de la Canal & Pinedo, 2018). In fact, EVs were visualized in the secondary phloem and xylem of woody plants and a role in the transport of glucanases, which would be involved in cell wall remodelling was suggested (Chukhchin et al., 2019). Likewise, the analysis of yeast mutants compromised in the biogenesis of EVs and in cell wall building also suggests a potential role for EVs in cell wall remodelling (Childers et al., 2019; de Toledo Martins et al., 2019; Zhao et al., 2019). So, this putative function of EVs may not be exclusive to plants but rather common across all cell‐walled organisms.

The functions of EVs *in planta* open novel opportunities for practical applications. Emerging ideas include the modulation of the plant immune system (Rybak & Robatzek, 2019) or their direct involvement in delivering defense proteins, antimicrobial compounds and sRNA cargos which can be transferred to fungi, although these results are debated (Rutter & Innes, 2020). Nevertheless, possibilities for EV content and release manipulation suggest the exciting avenue of using engineered EVs as agrochemicals for crop protection. In addition, plant EVs (and PDNVs) have also been suggested as potential actors in human therapies. Indeed, one recent paper has shown that apoplastic EVs can be taken up by ovarian cancer cell line (Liu et al., 2020) and few studies have reported therapeutic impact of PDNVs against tumour cell growth, colitis and infectious microbes (Ju et al., 2013; Kocak et al., 2020; Raimondo et al., 2015; Sundaram et al., 2019; Teng et al., 2018; Zhuang et al., 2016). Despite some recent debate about the reproducibility of PNDV miRNAs as regulators of mammalian cell targets (Campbell, 2020), the field is wide open for further studies of both PDNVs and true plant EVs in therapeutics.

## 6 CONCLUSIONS

Plant EVs research is still in its infancy, requiring much more data to substantiate claims about the types and roles of EVs as well as possible therapeutic applications in humans. The expected expansion of plant EV research must be firmly founded, though, on rigor and standardisation of nomenclature and procedures for purification and characterization of nanoparticles, both for natural EVs and PDNVs. Increased and rigorous data collection are expected to identify EV molecular content of proteins, lipids, and RNAs and discovery of long‐awaited EV markers. These markers are urgently needed to allow the plant field to better establish the roles of EVs in crop immunity and infection regulation as well as in biomedicine. The potentially tremendous impact of these numerous applications represents an amazing opportunity for the field of plant EVs.

Seen through the lens of rigor and standardization, the future of plant EVs is bright!
